# Empowering Patients and Supporting Health Care Providers—New Avenues for High Quality Care and Safety

**DOI:** 10.3390/ijerph18189438

**Published:** 2021-09-07

**Authors:** Isolde Martina Busch, Michela Rimondini

**Affiliations:** Department of Neurosciences, Biomedicine and Movement Sciences, University of Verona, 37134 Verona, Italy; isoldemartina.busch@univr.it

A large body of research suggests that establishing and strengthening patient–provider relationships, characterized by transparency, respect, trust, and empathy, is highly beneficial for patients, their caregivers, and healthcare providers [1,2,3,4,5]. However, due to disintegrated care and work pathways, staff reduction, and time pressures, the human aspects in the medical setting are often overlooked or disregarded [5,6,7]. Indeed, patients whose individual needs are underestimated may feel disrespected, disempowered, and dissatisfied with their care [5,8,9,10,11]. Moreover, healthcare providers are primarily assessed by their professional performance and are often under excessive work pressures, which can result in emotional distress and burnout [5,7,12,13].

The current COVID-19 pandemic has negatively influenced the psychological well-being of the general public, of patients, and healthcare providers in particular [14,15,16,17,18,19], worsening the above-described trend and emphasizing the urgent need for an integrative approach to healthcare that empowers patients, supports the healthcare workforce, and enhances public health efforts.

Following this rationale, this Special Issue covers high-quality, timely research on person-centered care, patient–physician communication, health literacy, healthcare providers’ professional and emotional well-being, and patient safety culture. Its overall structure takes the form of nine papers, comprising qualitative and quantitative studies, systematic reviews, and a theoretical paper, which make for compelling reading.

With regard to advancing patient care, the feasibility study by Lewandowski et al. [19] describes the process of implementing person-centered care for pediatric patients with moderate scoliosis in a Polish rehabilitation hospital. Using semi-structured interviews and applying content analysis, the results showed that patients and their families, as well as healthcare providers, perceived the approach as beneficial and feasible. The authors suggest that this type of care might even lead in the long run to reduced healthcare expenses and better quality of life for patients. Similarly, Madani Larijani et al. [20] promoted patient engagement by involving patients in the development of a tool tackling the overuse of lower back pain imaging. According to Madani Larijani et al. [20], the patient-oriented prescription pad, which gives information on the technique’s risks and benefits, may help empower patients by equipping them with knowledge and increasing patient involvement in medical decision making and fostering the patient–physician relationship. Van der Velden et al. [21] focused on a particularly difficult issue that may arise in patient–physician relations, namely, discussing prognoses for patients with advanced cancer who seek another oncologist for a second opinion. The qualitative study, investigating the conversations between patients with advanced cancer and their consulting oncologists, showed that oncologists were cautious in providing prognosis despite patients’ implicitly communicated cues and explicitly posed questions. A useful approach for tackling challenging topics in healthcare conversations, such as cancer prognosis, is described in the study by Borghi et al. [22]. The Italian Program to Enhance Relations and Communications Skills (PERCS-Italy), established in two hospitals in Milan in 2008, has been shown to improve healthcare providers’ self-reported professional skills, decrease anxiety, and enhance self-confidence during difficult conversations with patients.

One study in this Special Issue did not directly concentrate on patient engagement but on community engagement and empowerment and public health actions. Against the background of the global COVID-19 pandemic and its accompanying infodemic, Rubinelli et al. [23] aimed to detect targets for fostering critical health literacy in the population. Following argumentation theory, the authors performed a textual analysis of instances of health information and suggested that strengthening individuals’ skills in identifying and assessing arguments might be the path forward to empower the public in critically appraising health information.

The cross-sectional study by Gilles et al. [24] also focused on the current pandemic, assessing the professional well-being and their intent to stay by health care workers who were reassigned during the first pandemic wave in Switzerland. The findings indicated that not being able to choose to accept or decline a reassignment to the frontline of the COVID-19 crisis might negatively affect healthcare workers’ well-being and increase turnover intention. However, the responsiveness of hospital management was found to moderate this relationship. Thus, the authors call for hospitals and healthcare institutions to be responsive to healthcare workers and offer solutions adapted to their needs, such as flexible schedules and extraordinary leaves. Another study revolving around healthcare providers’ emotional well-being and mental health is a systematic review by Busch et al. [25], which gives an overview of the existing psychological support resources for second victims in the aftermath of adverse events. The article points out the lack of program availability, in particular outside the United States, and underlines the necessity of establishing programs that provide not only psychological first aid but also medium- and long-term support to enhance individual and, eventually, system resilience. The authors also recommend expanding such programs to healthcare providers suffering from other stressful clinical events, such as workplace violence and pandemic-related traumatic experiences and emotional distress.

Another group deserving special attention and support is the next generation of healthcare providers. Given that patient safety is crucial for delivering high-quality care [26], aspiring healthcare providers should be supported in acquiring adequate patient safety skills [27,28,29]. To better understand patient safety attitudes among young healthcare workers and healthcare profession students, Tocco-Tussardi et al. [30] synthesized the existing literature and concluded that this population shows overwhelmingly positive attitudes in certain domains, such as teamwork climate and error inevitability but more negative perceptions in other areas, such as safety climate and disclosure responsibility. The authors urge health professions educators and institutions to create a learning culture by integrating patient safety education and training in the curricula of aspiring healthcare professionals and to guarantee that the influential hidden curriculum better reflects the elements of the explicit curriculum. Finally, Fichera and colleagues [31] draw our attention to the still existing gender inequalities in clinical and academic radiology and underline the importance of empowering female radiologists, which may lead to improved professional and emotional well-being and better performance of the entire healthcare system.

Encompassing various articles, the Special Issue “Empowering Patients and Supporting Health Care Providers—New Avenues for High Quality Care and Safety” reflects a wide spectrum of different topics regarding patient care and clinician well-being.

We hope that this collection of original contributions may give rise to exciting new research questions in the field of healthcare quality and safety and accelerate progress towards a resilient and thus high-performing healthcare system that actively engages patients and their caregivers and effectively supports healthcare providers from the very beginning of their career.

## Data Availability

Not applicable.
